# Socio-demographic, clinical and therapeutic features of patients treated for schizoaffective disorder using cannabis

**DOI:** 10.1192/j.eurpsy.2023.1419

**Published:** 2023-07-19

**Authors:** W. Bouali, W. Haouari, S. Brahim, N. Faouel, L. Zarrouk

**Affiliations:** Psychiatrie, Faculty of Medicine of Monastir, Mahdia, Tunisia

## Abstract

**Introduction:**

Psychotic disorders were formerly associated with cannabis use. It could accelerate the course of the illness and thus, constitutes a severity factor in terms of prognosis.

**Objectives:**

To define the socio-demographic, clinical and therapeutic profiles of patients suffering from schizoaffective disorder (ASD) and who are consuming cannabis.

**Methods:**

A retrospective study of 16 patients diagnosed with ASD, who were hospitalized at the psychiatric department of Tahar Sfar Mahdia’s hospital, and whose toxicology test results during the hospitalization came back positive for tetrahydrocannabinol.

**Results:**

16 patients were gathered, all male, the average age was 26 years. The average age of first hospitalization was 25 years, 41.9% were unemployed. 76.3% of our sample were single. Three quarters of patients were hospitalized without consent. The average hospital stay was 30.33 days. Our patients had required during their stay an average dosage of antipsychotic, equivalent to chlorpromazine, of 752.42 +/- 342.79 mg. The average scores of psychometric scales were: BPRS = 55.72 +/- 14.11, SAPS = 41.5 +/- 14.80 and 42.11 +/- 18.88.

**Conclusions:**

Currently, it is recognized that prolonged use of cannabis is an exogenous risk factor. The association between cannabis and schizoaffective disorder may amend the treatment modalities. It requires, thereby, an integrated and simultaneous treatment of schizophrenia and addictive behavior.

**Disclosure of Interest:**

None Declared

